# HaCaT Keratinocytes Response on Antimicrobial Atelocollagen Substrates: Extent of Cytotoxicity, Cell Viability and Proliferation

**DOI:** 10.3390/jfb5020043

**Published:** 2014-05-08

**Authors:** Jorge López-García, Marián Lehocký, Petr Humpolíček, Petr Sáha

**Affiliations:** 1 Centre of Polymer Systems, Polymer Centre, Tomas Bata University in Zlin, T.G.Masaryk Sq. 5555, 76005 Zlin, Czech Republic; E-Mails: vextropk@gmail.com (J.L.-G.); humpolicek@ft.utb.cz (P.H.); saha@utb.cz (P.S.); 2 Polymer Centre, Faculty of Technology, Tomas Bata University in Zlin, T.G.Masaryk Sq. 275, 76272 Zlin, Czech Republic

**Keywords:** atelocollagen, antibacterial surface, cytotoxicity, cell proliferation, MTT assay

## Abstract

The effective and widely tested biocides: Benzalkonium chloride, bronopol, chitosan, chlorhexidine and irgasan were added in different concentrations to atelocollagen matrices. In order to assess how these antibacterial agents influence keratinocytes cell growth, cell viability and proliferation were determined by using MTT assay. Acquired data indicated a low toxicity by employing any of these chemical substances. Furthermore, cell viability and proliferation were comparatively similar to the samples where there were no biocides. It means that regardless of the agent, collagen-cell-attachment properties are not drastically affected by the incorporation of those biocides into the substrate. Therefore, these findings suggest that these atelocollagen substrates enhanced by the addition of one or more of these agents may render effectiveness against bacterial stains and biofilm formation, being the samples referred to herein as “antimicrobial substrates” a promising view in the design of novel antimicrobial biomaterials potentially suitable for tissue engineering applications.

## 1. Introduction

As a biomaterial for industrial application, collagen has been widely used in many fields, such as cell cultures, cosmetics, foods and medicines [1]. With regard to medical applications, this protein possesses an excellent biocompatibility, innocuousness and biodegradability. Due to these reasons, this matrix is deemed as a primary resource in biomedical applications and one of the most useful biomaterials that may be prepared in different forms, such as blocks, films, gels [2], pellets, sheets [3], sponges [4] and tubes [5].

Skin comprises essentially three types of cell: keratinocytes, melanocytes and fibroblasts. It is foreseen through wound healing, transplantation and cell culture studies that HaCaT cells may be used as an *in vitro* model for highly proliferative epidermis in tissue engineering. The spontaneously immortalised HaCaT cell line has been a widely employed keratinocyte model due to its ease of propagation and near normal phenotype, but protocols for differentiation and gene delivery into HaCaT cells are extensively found in the literature [6,7,8].

A serious difficulty in tissue replacement is biofilm formation, which is responsible for infections over the treated areas. Several implants have to be removed by their poor performances. Indeed, infections are the foremost common cause of biomaterial implant failure in medicine [9,10,11,12]. Different types of polymers are often sterilised via dry/wet heating or irradiation. Nevertheless, these materials may get contaminated by microbes once they are exposed to atmospheric conditions again. Hence, the preparation of anti-infective polymeric implants is a powerful way to overwhelm this problem [13,14,15,16]. One method to develop these kinds of materials is by adding organic or inorganic antimicrobial agents in the polymers during processing [17,18,19,20,21].

**Figure 1:**
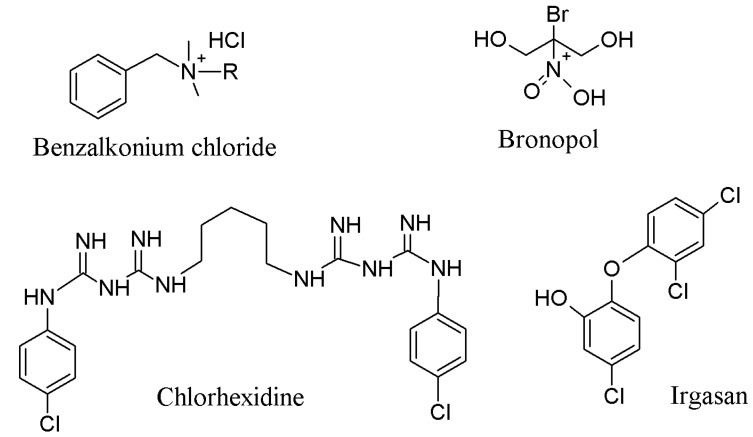
Chemical structure of employed antibacterial agents.

Antimicrobial agents are substances able to counteract or inhibit microorganisms [22]. Benzalkonium chloride is a quaternary ammonium compound, which is one of most used and known synthetic biocides in pharmaceutics [23,24]. Bronopol (2-bromo-2-nitropropane-1,3-diol) is a chemical compound which has a low toxicity in mammals and a high activity against bacteria, being a popular preservative in many personal care products as shampoos, colognes, deodorants, facial tissues, shaving creams amongst others personal hygiene products [25,26] Chlorhexidine, (1,1-hexamethylene bis[5-(4-chlorophenyl)biguanide]), is recognised as a chemical antiseptic by its effectiveness on both gram-positive and gram-negative bacteria. It is the active ingredient in oral rinses, skin cleansers, topical solution for veterinary use and, in small quantities, it is used as a preservative [27,28]. Another biocide, that holds immediate long term antibacterial efficiency and marginal toxicity in clinical use, is Irgasan (5-chloro-2-(2,4-dichlorophenoxy)phenol). The chemical structures are displayed in Figure 1 [29].

**Figure 2:**
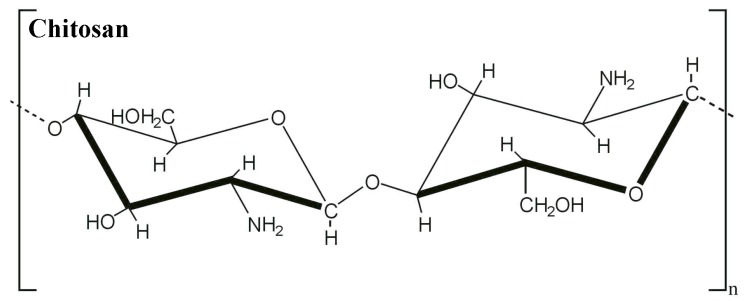
Chitosan chemical structure.

Chitosan is a deacetylated product of chitin, which is produced by chitin alkaline deacetylation (Figure 2) and this product has properties, such as antimicrobial activity and low toxicity [30]. Besides, it is highly synthesised because chitin is the second-most abundant biopolymer in nature. It is found in the cell walls of fungi, the exoskeletons of arthropods, insects, molluscs and cephalopods [31].

On account of the high impact of nosocomial infections in hospitals, the state of art in antimicrobial polymers is quite extensive and well documented. Nonetheless, there are few publications which have been committed to study the incorporation of the above-mentioned antibacterial agents either into biopolymers bulk or in their surfaces. Therefore, the main focus of this contribution is aimed at the addition of these chemical substances onto collagen matrices and to evaluate how those may influence keratinocyte cell response on atelocollagen films by means of cytotoxicity and cell proliferation studies. The findings of this research may help to strengthen knowledge on fields, such as antimicrobial biopolymers, human cell growth and tissue engineering.

## 2. Results and Discussion

### 2.1. Results

**Figure 3:**
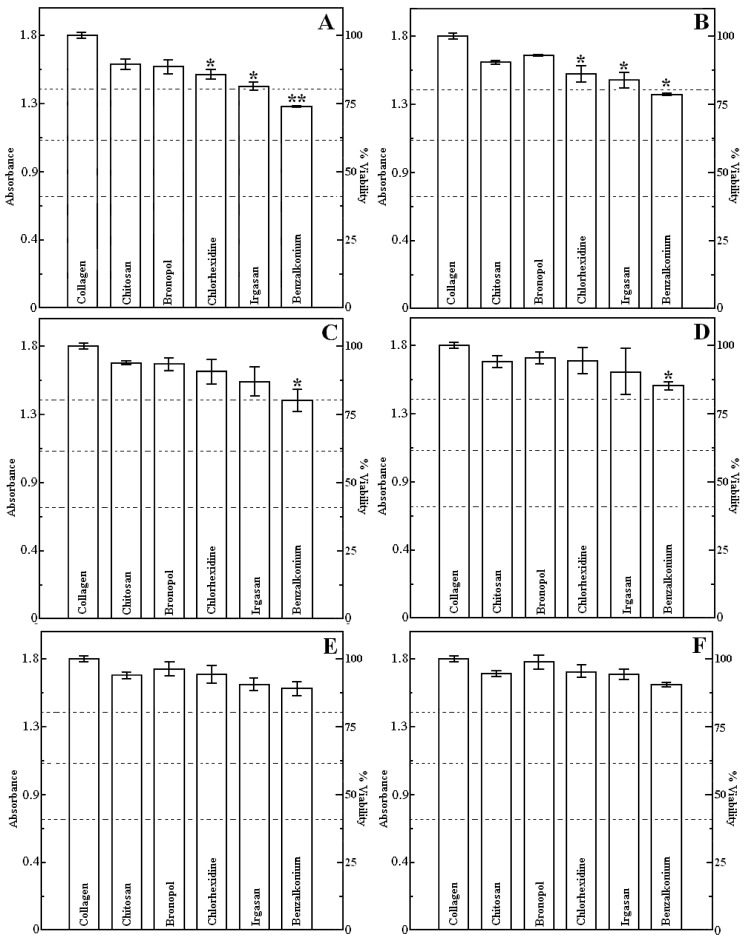
Effect of various concentrations of target compounds on HaCaT cell viability. Cell line seeded on atelocollagen matrix with concentration of agent (**A**) 2.0% (w/w); (**B**) 1.0% (w/w); (**C**) 0.5% (w/w); (**D**) 0.2% (w/w); (**E**) 0.1% (w/w); (**F**) 0.02% (w/w) determined by MTT assay (the error bars depict standard deviations and dashed lines define cytotoxicity ranges: non-cytotoxicity > 80%; weak > 60%; moderate > 40%; strong < 40%). *p < 0.05 and **p < 0.01, compared with pristine atelocollagen film.

The extent of cytotoxicity from every single concentration of antibacterial agent was quantified as a percentage of cell viability including the absorbance values obtained to each system (Figure 3A–F). Pursuant to ISO 10993-5, percentages of cell viability above 80% are considered as non-cytotoxicity; within 80%–60% weak; 60%–40% moderate and below 40% strong cytotoxicity respectively [32]. It may be seen in the histograms that these percentages were high and consequently, these substances were innoxious no matter the concentration that was used. The viability range was within 74%–99% and merely two samples of benzalkonium chloride 2.0% and 1.0% presented a weak cytotoxicity. The lowest value was found on collagen-benzalkonium chloride 2.0%, whilst the highest one was for the matrix with bronopol 0.02%. Figure 3A–C depicts yields over 80%. For instance, the highest concentration (Figure 3A) had a set of values within 74%–89%; it may be observed that the substrates endowed with agents at this concentration exhibited the smallest viability rates and the maximum value did not even reach 90%. All the experiments performed with 1.0% of biocide overcame 80% of viability except the sample with benzalkonium chloride (Figure 3B 78–93). Figure 3C that corresponds to 0.5%, the percentages were between 80% and 94% and the specimens with bronopol, chitosan and chlorhexidine had viabilities over 90%.

On the other hand, the lower concentrations (Figure 3D–F) describe yields above 90% with two exceptions, benzalkonium chloride 0.2% and 0.1%. (Figure 3D 85%–95%). The histograms of Figure 3E disclose that solely the matrix with collagen-benzalkonium chloride 0.1% did not attain 90% and the viability range for this concentration was within 89%–96%.

With reference to the samples with the minimum concentration, which is 100 times lower than the most concentrated one, 0.02% (Figure 3F). These showed the highest percentages 90%–99%. The data demonstrates that these biocides do not drastically inhibit the viability of HaCaT keratinocytes cell line. It may be noticed the increase of these values as concentration decreases, which points out the intrinsic connection between cell growth and amount of cytotoxic drug [33,34,35,36]. Statistically speaking, seven samples were found significantly different at a significance level of 0.05 (chlorhexidine 2.0% and 1.0%; irgasan 2.0% and 1.0%; benzalkonium chloride 1.0%, 0.5% and 0.2%) and one (benzalkonium chloride 2.0%) as very significant.

**Figure 4:**
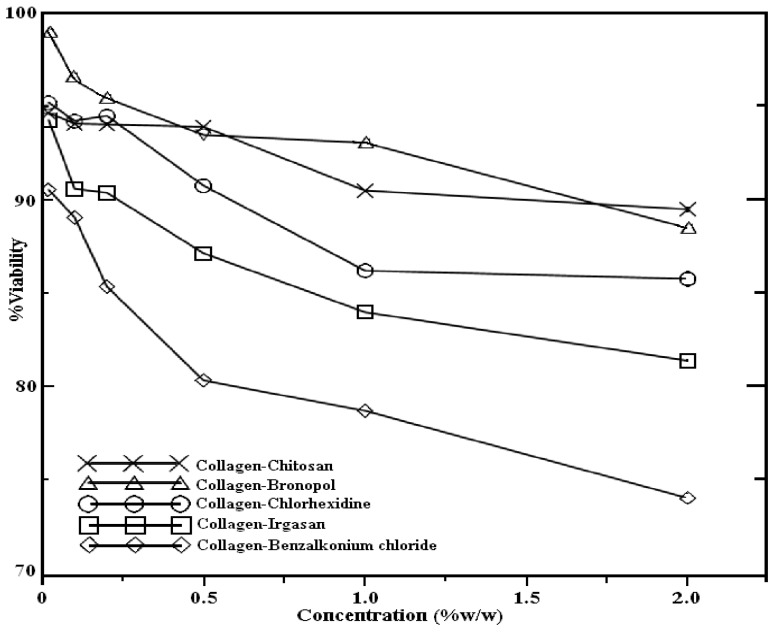
Concentration-viability curve of studied agents on HaCaT keratinocyte cell line.

Figure 4 seeks to give other view of the results on Figure 3 by showing the dependence of cell viability on concentration of agent in scatterplots instead of histograms. It shows the percentage of viability with respect to this variable. It reveals a fall in cell viability by increasing concentration. In general, all the plots have the same pattern. Chitosan is a particular case, because its curve evinces a plateau followed by a decreasing trend at the highest concentrations (1.0% and 2.0%). 

**Figure 5:**
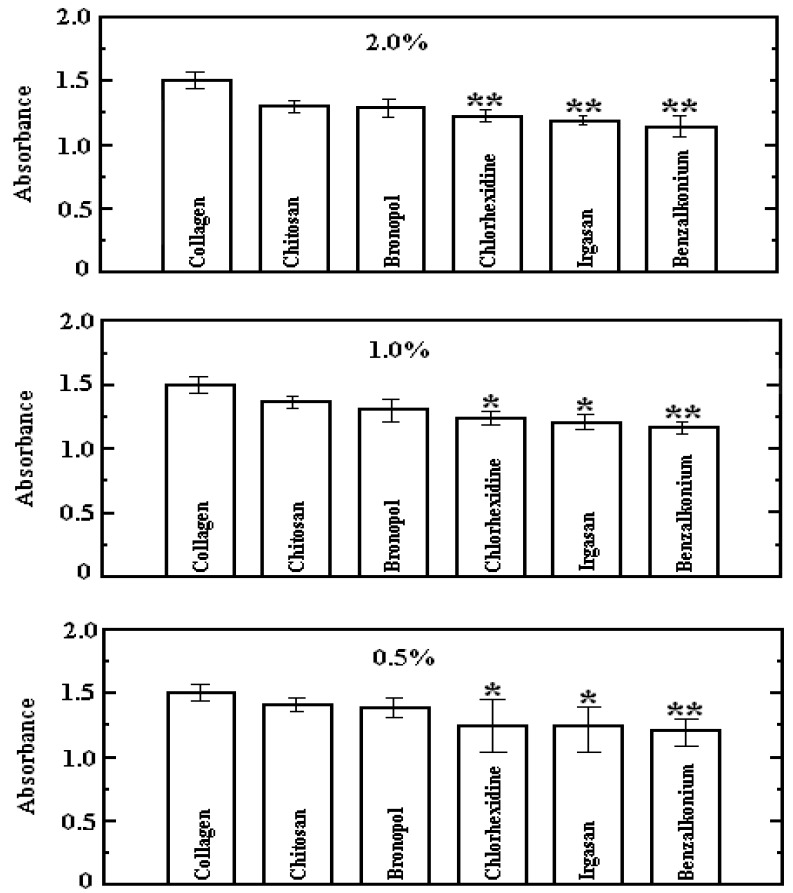
Comparison of HaCaT cell growth upon atelocollagen films with and without bactericides determined by measuring absorbance of Formazan product by MTT assay at 570 nm (the error bars signify standard deviation). * *p* < 0.05 and ** *p* < 0.01, compared with free of biocide film.

HaCaT cell proliferation on the substrates with and without biocides is given in Figure 5. It was ascertained that cell attachment marginally diminished, as reflected by the spectrophotometric data of each test, where all the absorbances corresponding to the added-agent samples were smaller than the pristine ones. It may be noticed that only the substrates endowed with chitosan and bronopol did not show a statistical significance. Conversely, all the samples with chlorhexidine, irgasan and benzalkonium chloride have statistical significance and in five cases, these were considered as very significant ones, chlorhexidine 2.0%; irgasan 2.0%; benzalkonium chloride 2.0%, 1.0% and 0.5% respectively.

**Figure 6:**
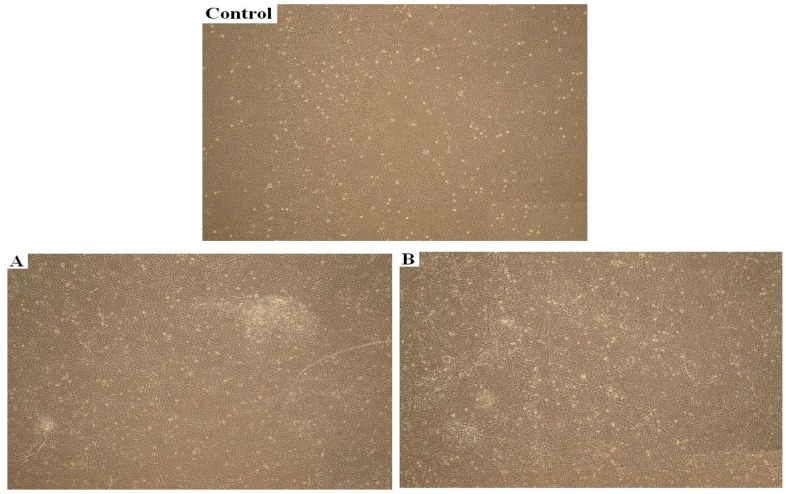
Photomicrographs of Human skin HaCaT keratinocytes in culture upon two atelocollagen films compared with control: (**A**) Atelocollagen without biocides; (**B**) An endowed film with cell viability above 80% (non-cytotoxic).

These results were also qualitatively supported by the photomicrographs in Figure 6, where the extent of cell adhesion on two non-cytotoxic substrates may be compared to a control specimen. As far as surface morphology is concerned, the images show a vast amount of cell aggregates in form of ripple-like areas adhered on the surfaces, with the typical keratinocyte cell shape.

### 2.2. Discussion

As may be noted across this study, chitosan, bronopol and chlorhexidine have lower inhibition in comparison with irgasan and benzalkonium chloride, which are the strongest ones in all the cases. As well as it is important to emphasise the importance of pH and solubility on the yield of each agent, since some of these bactericides do not possess the same effectiveness and stability in acid solution. E.g., chitosan, bronopol and benzalkonium chloride are readily soluble and stable in water and acid solution. In contrast to irgasan, which is slightly soluble in this medium and chlorhexidine is sparingly soluble and unstable in acid pH [37,38,39,40,41]. As described in material section, the mixtures were prepared by using a stirring machine for 4 h at 1000 rpm. When the biocide is soluble in the employed solvent, a good dispersion (with cohesive character) and distribution of the agent into the mixture is obtained. Contrariwise whether the substance is moderately soluble, the mixture is not uniform having loss of agent during processing, and thus the final concentration is different and lower than the one that was intended [42]. It means that irgasan, which has low solubility under the experimental conditions is even able to suppress HaCaT cell growth. Whilst, despite bronopol and chitosan, are soluble do not represent a serious risk to the viability of this cell line.

It is worth mentioning that for the studied samples preparation, the solvent (acetic acid 0.1 M) had to be completely evaporated, since HaCaT as well as most cells require pHs around 7.0 and the permanent control of pH is essential for optimal culture conditions [43,44].

Concerning the chosen method to estimate the extent of cytotoxicity, MTT is a rather cost-efficient colorimetric technique, where the measurement strictly depends upon live cells, since the tetrazolium salt 3-(4,5-dimethylthiazol-2-yl)-2,5-diphenyltetrazolium bromide is reduced to formazan product exclusively by mitochondrial succinate dehydrogenase enzyme in the mitochondria of viable cells. Hence, this assay ensures a good approximation in the study of cell viability and proliferation in cell culture, where dead cells do not participate as interfering species. There are other factors that may induce the reduction of MTT, such as times of incubation, age of culture, media poverty in glucose, and reagents stability [45].

Cell proliferation under chitosan medium was higher than under the other media followed by bronopol, chlorhexidine, irgasan and benzalkonium chloride respectively. This information coincides with the cytotoxicity assay results, where the studied biocides performed in similar way in both experiments (Chitosan, bronopol and chlorhexidine have lower inhibition ability than irgasan and benzalkonium chloride).

Although the mechanisms of HaCaT cell adhesion and proliferation on different substrates are still unclear, it is well-established that HaCaT keratinocytes proliferate better on rough, porous and hydrophilic scaffolds. The cases of atelocollagen-chlorhexidine and irgasan substrates are a proof of that, since these agents are toxic *per se* to this cell line and also because of their low solubility in the medium. These substances may alter hydrophilicity, diminishing cell adhesion and proliferation [46,47].

In culture, keratinocyte cells behave in a similar way they do *in vivo*, where cells migrate towards the air interface to form the epithelial surface. Epidermal substitutes require minimum two weeks to expand keratinocytes population. For these reasons, it is necessary to pay heed to the stability of keratinocytes attachment [48]. In that respect, the findings indicate that after four days in culture these substrates hold low marginal toxicity, as well as suitability for cell proliferation. In fact, the MTT assay was performed after 8 and 15 days showing no effect on the cell growth for and all the samples with a concentration lower than 0.5% expect for the system with benzalkonium chloride. In contrast, 2.0% and 1.0% exhibited a decrease in cell growth by the passage of time. This drop was under 80% of cell viability in the case of chlorhexidine, irgasan and benzalkonium systems, but none case reached the moderate strong cytotoxicity range, which is within 60%–40%. The specimens endowed with chitosan and bronopol 2.0% and 1.0% showed a slight alteration and all their systems kept as non-cytotoxicity regardless of the experimentation time. 

Surface adherence is a natural tendency, which is inherent to bacteria and other microorganisms. It has four basic steps: adhesion, colonization, formation and the subsequent bacterial biofilm growth, which is independent of the substrate. Biofilms act as defence mechanism against external agents; in consequence, the aim of any antimicrobial materials is at preventing bacterial adhesion and colonization, which are prerequisites to biofilm formation [49]. It is known by literature that benzalkonium chloride, bronopol and chitosan hinder the adhesion of gram-positive strain, but do not behave satisfactorily against gram-negative bacteria [50,51]; chlorhexidine and irgasan are efficacious against both strains [52,53]. According to biothermodynamic studies, bacteria may attach to both hydrophobic and hydrophilic surfaces; notwithstanding, hydrophobic surfaces are colonised faster than hydrophilic ones [54,55]. This feature rises in importance, since the studied substrates are highly hydrophilic, which is favourable to HaCaT cell adhesion but not to bacterial colonization.

The described phenomenon is largely surface specific and affects material functionality leading to loss of physical and mechanical properties [56,57]. The overall outcome shows that by using a proper biocide concentration cell viability and proliferation are barely impacted keeping the collagen-cell-attachment properties almost steady (Cell viability rates beyond 80%). Consequently, these atelocollagen substrates enhanced by the addition of one or more of these agents may render effectiveness against bacterial stains and biofilm formation, being a promising view in the design of novel antimicrobial biomaterials potentially suitable for tissue engineering applications, since atelocollagen is indeed prospective scaffold for cell growth purposes, which also has the advantage of being eliminated by degradation processes similar to the metabolism of endogenous collagen. 

## 3. Experimental Section

### 3.1. Materials

Atelocollagen emulsion from bovine Achilles tendon (pH 3.5), which contains 1.4% of atelocollagen was supplied by Vipo A.S (Partizánske, Slovakia). Acetic acid 99% was obtained from Penta (Prague, Czech Republic). Bronopol (2-bromo-2-nitropropane-1,3-diol) C_3_H_6_BrNO_4_ 98% was purchased from Fluka (St. Louis, MO, USA). Benzalkonium chloride with a predominant formula of C_12_H_25_N(CH_3_)_2_C_7_H_7_Cl 98%; chitosan 98%; chlorhexidine (1,1-hexamethylene bis[5-(4-chlorophenyl)biguanide]) C_22_H_30_Cl_2_N_10_ 98%; irgasan 5-chloro-2-(2,4-dichlorophenoxy)phenol C_12_H_7_Cl_3_O_2_ 97% and Dimethyl sulfoxide (DMSO) were provided by Sigma-Aldrich (St. Louis, MO, USA). The reagents in this study were used as received without any further purification. Tissue culture dishes of 40 mm diameter and individual wells of 96-well were commercially acquired from TPP (City, Switzerland). Vybrant^®^ MTT cell proliferation Assay kit V-13154 was purchased from Invitrogen Corporation (Carlsbad, CA, USA).

### 3.2. Preparation of Collagen-Antibacterial Agent Substrates

Five mother mixtures of atelocollagen with each antibacterial agent (2.0% weight of agent/weight collagen) were prepared by dissolving these compounds in 0.1 M water solution of acetic acid to obtain a 0.1% weight of collagen/weight of solution, using an IKA RCT stirring machine (IKA^®^ works, Inc, Staufen, Germany) for 4 h at 1000 rpm. Less concentrated solutions (1.0%, 0.5%, 0.2%, 0.1% and 0.02%) were prepared by simple dilution. Each group of samples was casted on tissue culture dishes and the solvent was evaporated at ambient conditions for three days. Thin films of pristine atelocollagen were prepared and used as experimental blanks.

### 3.3. HaCaT Cell Incubation

Human immortalised non-tumorigenic keratinocyte cell line HaCaT, (Ethnicity, Caucasian; Age, 62 years; gender, Male and tissue, skin) were supplied by CLS Cell Lines Service (Eppelheim, Germany). Dulbecco’s modified eagle medium, contains 4.5 g/L D-glucose, L-glutamine, and 110 mg/L sodium pyruvate (DMEM; Invitrogen) supplemented with 2 mM L-glutamine, 10% foetal bovine serum (FBS) and penicillin-streptomycin (100 U/mL-0.1 mg/mL) was used as a culture medium (Biotech Inc., Oklahoma City, OK, USA). Cells were incubated at 37 °C for 24 h with 5% CO_2_ in humidified air.

### 3.4. Evaluation of Cytotoxicity (in-Vitro)

#### 3.4.1. Extract Preparation

The substrates obtained above were extracted according to ISO 10993-12 [58] in the ratio of 0.1 g of the films per 1.0 mL of culture medium in chemically inert closed containers by using aseptic techniques. Each extract was incubated in DMEM medium at 37 ± 1 °C with stirring for 24 h [59].

#### 3.4.2. Cell Viability of HaCaT

All cells in the exponential growth phase were seeded in a concentration of 1 × 10^5^ cells/mL onto the substrate extracts (2.0%, 1.0%, 0.5%, 0.2%, 0.1% and 0.02%). Cell viability as indicator of cytotoxicity was determined after 4 days of culture by MTT assay. Absorbances were recorded by using a Sunrise microplate ELISA reader at 570 nm (Tecan group, Männedorf, Switzerland), and all determinations were performed in quadruplicate [60,61].

### 3.5. Cell Proliferation Test

HaCaT cell proliferation on thin films with the following specifications: collagen-benzalkonium chloride, collagen-bronopol, collagen-chitosan, collagen-chlorhexidine and collagen-irgasan 2.0%, 1.0% and 0.5% was determined after 4 days of culture by MTT assay. A volume of 10 μL of 12 mM MTT was taken for cell incubation performed at 37 °C for 4 h in the darkness. Thereafter, the media were decanted and washed with phosphate-buffered saline solution (PBS). The produced formazan salts were dissolved with dimethylsulphoxide (DMSO) and its concentration was measured in a spectrophotometer at 570 nm [62]. The photomicrographs were taken by using an inverted phase-contrast microscope Olympus CKX41 (Olympus, Münster, Germany) with an optical zoom of 40-times.

### 3.6. Statistical Analysis

All data were presented as the mean value ± standard deviation (SD) of each sample. Statistical comparisons were performed using Student’s t-test with a confidence level of 95% (*p* < 0.05) considered statistically significance and 99% (*p* < 0.01) considered very significant.

## 4. Conclusions

This contribution delved into the incorporation of bactericides to atelocollagen matrices. The mixtures of atelocollagen with benzalkonium chloride, bronopol and chitosan are uniform and stable. Cell viability of HaCaT is barely altered by the presence of these substances (74%–99%). Only in eight from thirty samples the cell viability was statistically lower than that found on the substrates without biocides. It means that any of these substrates provides an appropriate environment for this cell line. Thus, the studied samples are perfectly apt for keratinocyte cell growth. Cytotoxicity is concomitant to concentration and depends upon each agent. Bronopol and chitosan arise as the less hazardous to this cell line having percentages of viability beyond 85% with a negligible cytotoxicity at lower concentrations; whereas irgasan and benzalkonium chloride manifest more power of inhibition with the highest rates of cytotoxicity throughout the study. This inhibition pattern might be observed in both cytotoxicity and proliferation experiments and confirmed by statistic.
